# Optical in-memory computing using laser array

**DOI:** 10.1038/s41377-026-02338-x

**Published:** 2026-06-02

**Authors:** Omar Alkhazragi

**Affiliations:** Department of Electrical Engineering, King Fahd University of Petroleum and Minerals (KFUPM), Dhahran, 31261 Saudi Arabia

**Keywords:** Optical techniques, Lasers, LEDs and light sources

## Abstract

A new optical in-memory computing system based on an array of vertical-cavity surface-emitting lasers (VCSELs) has the potential to circumvent the Von Neumann bottleneck. The high modulation speed of the lasers in the array allows for fast computing and their high efficiency can enable edge computing in autonomous vehicles and drones. This efficient, highly scalable system was demonstrated to perform 900 million convolutions per second with 98% computing accuracy.

Optical computing has long been proposed as a pathway to overcome the limitations of conventional electronic computing, especially for demanding applications. One of the most important examples of these emerging applications is deep neural networks (DNNs), which are used in artificial intelligence (AI) systems for computer vision and reasoning. These systems rely on intensive computing that includes hundreds of millions of operations per second for low-latency applications. Therefore, implementing them using traditional computing on edge devices that have limited power and size has proven to be challenging. This is because traditional central computing units (CPUs) are limited by the Von Neumann bottleneck. Several optical computing systems have been proposed as an alternative approach to enable using DNNs on edge devices by avoiding using wires for information transport and by allowing a high level of parallelism of matrix-vector multiplication (MVM) operations.

Optical neural networks (ONNs) of different forms are already used for different applications^1,2^. Some of them rely on integrated chips by using devices such as Mach–Zehnder interferometers (MZIs)^3,4^ or micro-ring resonators (MRRs)^5,6^, while others operate in free space using diffractive optical elements (DOEs), spatial light modulators (SLMs), and digital micromirror devices (DMDs)^7,8^. The approaches based on photonic integrated circuits (PICs) face limitations in scalability due to the large size of interferometers and modulators. On the other hand, free-space systems are limited by the switching speeds of SLMs or DMDs, which prevents high clock rate operation. Therefore, to implement an ONN in edge devices that demand high computing speed and low latency, alternative designs that provide the needed processing speed with low power consumption are considered.

A recently published study aims to address this challenge by using an array of vertical-cavity surface-emitting lasers (VCSELs) for input activation and an SLM for reconfigurable weighting ^9^. The proposed scheme, shown in Fig. 1, consists of a VCSEL array, a DOE to perform spatial fanout of copies of the output of the laser array, an SLM whose pixels carry the weights, and a photodetector (PD) array. The detection is based on differential readout to allow for signed weights. The high modulation speed of the VCSELs, which is in the range of GHz, is sufficient to enable high clock rate computation. Moreover, unlike edge-emitting laser diodes, the surface emission of VCSELs allows for the easy formation and high-volume manufacturing of 2D arrays of lasers. This is a critical advantage that makes them ideal for massive parallelization. While the VCSEL array in the study consisted of 5 × 5 lasers, larger arrays are widely used in a variety of applications. The DOE creates 3 × 3 copies that are sent to the SLM. The pixels of the SLM can be programmed based on the required weights. The SLM is divided into 3 × 3 kernels, each operating on a copy of the 5×5 beams. The propagation of light through the SLM performs the multiplication, while the accumulation is done by summing the output beams on the detectors, which generate currents proportional to the aggregate of the summed-up beams. The high pixel count of SLMs (typically in the millions) is another key enabler for the system that makes it theoretically possible to perform millions of multiplications in parallel. To achieve the same level of parallelism in integrated systems would require a large chip size (given the relatively larger size of MZIs and MRRs compared to an SLM pixel) and a precise fabrication process to ensure high yield.

**Fig. 1:**
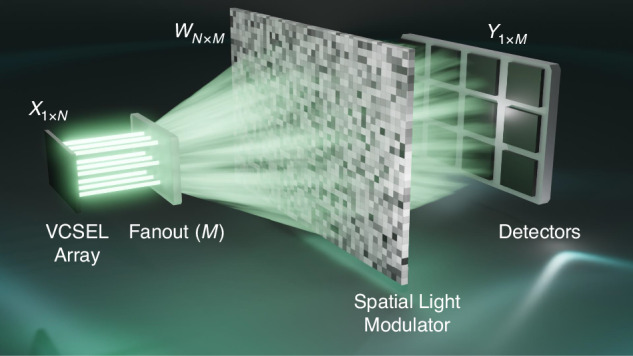
Optical computing using VCSEL arrays: an illustration of the optical computing setup

The fanout spatial time-of-flight optical neural network (FAST-ONN) system performance was benchmarked against demanding computer vision tasks in a you-only-look-once (YOLO) object classification system to identify cars. This is an example of an application where low latency is important for decision making in a short amount of time. Using the common objects in context (COCO) dataset to test the system, the area under curve (AUC) of the receiver operating characteristic (ROC) curve was 0.98. In addition, the FAST-ONN system supports “photonic reprogrammability,” enabling on-device training. The system successfully performed in-situ learning on 800 randomly selected images from the standard 10-class modified National Institute of Standards and Technology (MNIST) dataset, reaching 92.8% accuracy on a test subset. This capability is vital for edge devices that must adapt to rapidly changing environments without relying on energy-intensive data transfers to the cloud.

One of the most important advantages of the FAST-ONN design is its scalability. The VCSEL transmitters can be optimized to achieve higher modulation speeds, pushing clock rates into the tens of gigahertz range. This has the potential to improve the throughput of such systems by orders of magnitude. As the demand for more accurate AI models increases and as they continue to grow in size and complexity, the ability of FAST-ONN to provide high-speed matrix-vector multiplication operations under strict size, weight, and power (SWaP) limitations has the potential to open the door toward the next generation of intelligent, real-time edge sensors.
